# Distinctive Expression of Bcl-2 Factors in Regulatory T Cells Determines a Pharmacological Target to Induce Immunological Tolerance

**DOI:** 10.3389/fimmu.2016.00073

**Published:** 2016-03-01

**Authors:** Sarah Sharon Gabriel, Nina Bon, Jin Chen, Thomas Wekerle, Andrew Bushell, Thomas Fehr, Pietro Ernesto Cippà

**Affiliations:** ^1^Nephrology, Institute of Physiology, University of Zürich, Zürich, Switzerland; ^2^Division of Nephrology, University Hospital Zürich, Zürich, Switzerland; ^3^Transplantation Immunology, Department of Surgery, Medical University of Vienna, Vienna, Austria; ^4^Transplantation Research Immunology Group, Nuffield Department of Surgical Sciences, University of Oxford, Oxford, UK; ^5^Department of Internal Medicine, Cantonal Hospital Graubünden, Chur, Switzerland

**Keywords:** regulatory T cells, tolerance, apoptosis, ABT-737, Bcl-2, Bcl-XL, Mcl-1, transplantation

## Abstract

Distinctive molecular characteristics of functionally diverse lymphocyte populations may represent novel pharmacological targets for immunotherapy. The intrinsic apoptosis pathway is differently regulated among conventional and regulatory T cells (Tregs). Targeted pharmacological modulation of this pathway with a small molecule Bcl-2/Bcl-xL inhibitor (ABT-737) caused a selective depletion of effector T cells and a relative enrichment of Tregs *in vivo*. Treatment with ABT-737 resulted in a tolerogenic milieu, which was exploited to alleviate graft-versus-host disease, to prevent allograft rejection in a stringent fully MHC-mismatched skin transplantation model and to induce immunological tolerance in combination with bone marrow transplantation. This concept has the potential to find various applications for immunotherapy, since it allows pharmacologic exploitation of the immunomodulatory properties of Tregs without the need for cell manipulation *ex vivo*.

## Introduction

Maintenance of immunological tolerance requires functional CD4^+^CD25^+^FoxP3^+^ regulatory T cells (Tregs). Lack of Tregs results in fatal autoimmune lymphoproliferative disorder in mice and IPEX syndrome in humans (1, 2). The immunomodulatory potency of Tregs attracted great interest for immunotherapy in autoimmunity, allergy, and transplantation (3). The promising results obtained by adoptive transfer of Tregs in experimental models are currently being translated into clinical pilot studies of type 1 diabetes, graft-versus-host disease (GVHD), and solid organ transplantation (4, 5). However, individualized cell therapy requires specific infrastructure for Treg isolation and expansion, bears high costs and potential risks regarding stability, homogeneity, and fate of transferred Tregs (6–8). The growing understanding of the physiological mechanisms determining Treg development and survival might lead to alternative strategies to exploit their immunomodulatory properties directly *in vivo*.

Apoptosis is instrumental in shaping the immune cell repertoire and maintaining immune homeostasis. Pore formation in the mitochondrial outer membrane by Bax/Bak initiates an orchestrated dismantling of the cell by caspases (9). As pore formation is the point-of-no-return leading ultimately to cell death, this process has to be tightly regulated. Diverse internal and external signals, such as cytokine availability, T cell receptor (TCR) engagement, or costimulation, regulate the expression of pro- and antiapoptotic Bcl-2 family proteins in lymphocytes and thereby control the fate of the cell (10). Hence, the intrinsic apoptosis pathway is critically involved in controlling deletional tolerance in the thymus (11–13), selection of high-affinity clones (14), contraction of the conventional T cell (Tcon) pool after antigenic clearance (15), and maintenance of long-lived memory T cells (16). Importantly, survival of distinct T cell subpopulations depends on different Bcl-2 proteins. Naïve effector and memory T cells depend on Bcl-2 expression (16, 17), whereas IL-2-regulated Mcl-1 expression is particularly important for Treg homeostasis (18). Notably, Bcl-2 and Bcl-xL are dispensable for Treg survival, as demonstrated by the fact that FoxP3-specific deletion of Bcl-xL did not affect Treg survival (18).

This differential expression and dependency on Bcl-2 factors among lymphocyte subpopulations might be exploited as a novel target for immunotherapy. Particularly, small molecule inhibitors selectively targeting certain Bcl-2 family members might provide a novel pharmacological tool for immunomodulation through a selective depletion of lymphocyte subsets by apoptosis. The small molecule inhibitor ABT-737 binds Bcl-2, Bcl-xL, and Bcl-w proteins with high affinity, and Mcl-1 and A1 with much lower affinity (19). Thus, cell populations whose survival depends primarily on antiapoptotic Mcl-1 and/or A1 are resistant to ABT-737-mediated apoptosis (20, 21). We hypothesized that survival of Tregs should not be affected by Bcl-2 and Bcl-xL inhibition and that the previously reported lymphotoxic effect of ABT-737 might spare the Treg compartment (22, 23).

## Materials and Methods

### Mice

C57BL/6 (B6, H-2^b^), CBA (H-2^k^), BALB/c (H-2^d^), B6xCBAF1 (H-2^b/k^), B6.Cg-FoxP3^tm1Mal^/J (FoxP3-GFP, H-2^b^), and B6-Tg(FoxP3-DTR/EGFP)^23.3Spar^ (DEREG, H-2^b^) mice were housed under pathogen-free conditions at the University of Zürich and used for experiments at the age of 8–16 weeks, always in age- and sex-matched groups. FoxP3-GFP mice express eGFP under the control of the FoxP3 transcription factor (24) and were kindly provided by Bernard Malissen (Centre d’Immunologie de Marseille-Luminy, France). With aging, these mice seem to express higher percentage of Tregs. For this reason, FoxP3-GFP mice were always perfectly age-matched within one experiment. DEREG mice express a FoxP3 BAC-driven primate diphtheria toxin (DT) receptor fused to eGFP, allowing to specifically depleting FoxP3^+^ cells with DT administration (25). DEREG mice were purchased from The Jackson Laboratory, and B6, CBA, BALB/c, and B6xCBA F1 mice were purchased from Harlan Laboratories. All animal experiments were performed according to protocols approved by the local legal authority (Veterinary office, Canton of Zürich, Switzerland).

### Fluorescent-Activated Cell Sorting

Fluorescent-activated cell sorting analyses were performed with a FACS Canto II or LSR II Fortessa (Becton Dickinson). The following antibodies were used to stain 1 × 10^6^ cells of single cell suspensions of spleen or peripheral blood: CD4 (GK1.5) -PE, -APC, -AF700, CD8 (eBioH35.-17.2) -APC, B220 (RA3-6B2) -PE, CD25 (PC61.5) -PE/Cy7, -APC, F(ab’)2-FITC anti-rabbit IgG, FoxP3 (FJK-16s) -biotin, streptavidin–APC (all eBioscience), CD8 (53-6.7) -APC/Cy7 (all BioLegend), Mcl-1 (Y37), Bcl-2A1 (EP517Y) (both Abcam), Bcl-2 (3F11) -FITC (Becton Dickinson), and Bcl-xL (54H6) -Alexa488, Bim (polyclonal) (both Cell Signaling Technologies). For live-dead discrimination PI, 7-AAD or Zombie Aqua fixable viability kit (BioLegend) was used. Mice were bled sublingual and chimerism was analyzed in peripheral leukocytes at different time points after bone marrow transplantation (BMT), and donor-derived cells were identified by staining of donor H-2K^k^. From the signal measured in a positive control (CBA), the background signal of a negative control (B6) was subtracted and converted to be 100%. The same formula was then used to calculate the percentage of donor-derived cells in mixed chimeras. For intracellular staining, a FoxP3-staining buffer set (eBioscience) was used according to the manufacturer’s instructions.

### ABT-737 Sensitivity *In Vitro*

For *in vitro* experiments, ABT-737 (AbbVie Bioresearch) was dissolved in DMSO at a concentration of 5 mM and then further diluted in RPMI medium containing 10% fetal bovine serum, 100 U/ml penicillin, 100 μg/ml streptomycin, and 50 μM 2-mercaptoethanol. Either freshly isolated, *in vitro* IL-2- (R&D Systems, PeproTech) stimulated (3 days with 100 U/ml) or alloantigen-stimulated FoxP3-GFP splenocytes (see iTregs below) were plated in 96-well plates in culture medium at a concentration of 2.5 × 10^6^ cells/ml. Different amounts of ABT-737 or DMSO vehicle control were added to cells and incubated for 12 h at 37°C and 5% CO_2_. Cells were washed 3×, stained, and viability of CD4^+^(CD25^+^)GFP^+^ and CD4^+^(CD25^−^)GFP^−^ cell populations was assessed by PI or 7-AAD exclusion. Absolute cell counts per well were determined by the addition of absolute counting beads (Invitrogen). Surviving cell numbers of each population were normalized to the corresponding vehicle condition.

### *In Vitro* Antigenic Treg Stimulation

For Treg activation, mixed lymphocyte reactions (MLRs) were performed in 24-well plates. CBA splenocytes (stimulators) were depleted of T cells (CD3 microbeads), and FoxP3-GFP splenocytes (responders) were depleted of CD25^+^ cells by automatic magnetic cell separation according to manufacturer’s instructions (Miltenyi Biotec) and then plated at a 1:1 ratio at a concentration of 4 × 10^6^ cells/ml. Anti-CD154 antibody (50 μg/ml, MR1, Bio-X-cell) was added and cells were incubated at 37°C and 5% CO_2_. After 24 h of stimulation, 5 ng/ml TGF-β1 (R&D Systems, PeproTech) and 100 U/ml IL-2 were added to the cultures. After another 72 h of stimulation, all cells were pooled and plated in 96-well plates in order to test ABT-737 sensitivity (see above).

### *In Vivo* Antibody and Drug Treatments

Antibodies against CD154 (MR1), CD25 (PC61.5) (Bio-X-cell), and GITR [DTA-1, provided by Sakaguchi (26)] were diluted in PBS and injected i.p. as described below. DT (1 μg, Calbiochem) was administered by i.p. injection on two consecutive days (27). For *in vivo* application, ABT-737 was dissolved in polyethylene glycol, Tween 80, DMSO, and dextrose solution, and injected i.p. at 50 mg/kg. CsA (Enzo Life Sciences) was dissolved in a Cremaphor EL/ethanol solution, then diluted in HBSS, and injected s.c. at 10 mg/kg. Control mice were treated with the corresponding vehicles.

### Short- and Long-Term ABT-737 Treatment

For short-term ABT-737 treatment, mice received five ABT-737 injections from days −3 to −1, before their spleens were harvested and analyzed on day 0. For long-term ABT-737 treatment, mice received injections for 4 weeks every other day and were bled sublingual on days 0, 7, 14, 21, and 28.

### Skin Grafting

In all skin graft experiments, B6 or DEREG mice were recipients, CBA mice were donors, and BALB/c mice were third party controls. CBA and BALB/c are fully MHC-mismatched to B6/DEREG and among each other. For skin transplantations, recipient mice were anesthetized with ketamine/xylazine, pain treated with carprofen (NSAID), and shaved. Full thickness tail skin (about 1 cm^2^) of donors and third party controls was grafted to the back and considered rejected, when <10% of the graft remained viable (non-blinded).

### Donor-Specific Transfusion

In the donor-specific transfusion (DST) experiment, recipient mice were treated with a total of five injections of ABT-737/vehicle on days −3 to −1, and anti-CD154 antibodies on days 0, 6, 11, and 14 (0.25 mg each). On day 0, recipients received 1 × 10^7^ donor splenocytes in Media 199 containing 10 mM HEPES, 10 μg/ml DNAse, and 4 μg/ml gentamycin by tail vein injection. B6 recipients received anti-GITR (4 mg) antibodies on day 6, DEREG recipients DT on day 6/7, and skin was grafted on day 7.

### Bone Marrow Transplantation

The ABT-737 tolerance protocol was described in detail previously (28). The conditioning of mice consisted of ABT-737 and CsA treatment with a total of five injections on days −3 to −1, and one daily injection until day 11. Anti-CD154 antibodies (2 mg) were administered once 6 h before the transplantation of 25 × 10^6^ donor BM cells by tail vein injection on day 0 (in the same medium as described above for DST). Tregs were depleted with anti-CD25 antibodies (1.25 mg) or blocked with anti-GITR antibodies (4 mg) on day −1. Peripheral blood chimerism was monitored and 8 weeks after BMT all mice received skin grafts.

### Graft-Versus-Host Disease

On day 0, B6xCBA F1 recipient mice were lethally irradiated (900 cGy) with a Cs source. Six hours later, mice were transplanted with 1 × 10^7^ DEREG BM cells and 2 × 10^7^ DEREG splenocytes by tail vein injection (in the same medium as described above for DST), which corresponded to 5.7 × 10^6^ T cells in total. Furthermore, mice received cefazolin (antibiotic, 1 mg) after transplantation on days 0 and 2. ABT-737 and/or CsA treatment started on day −1 and was continued until day 10, and following doses were injected: day −1: 75 mg/kg ABT-737 and 15 mg/kg CsA; day 0: 100 mg/kg ABT-737 and 15 mg/kg CsA; days 1–3: 50 mg/kg ABT-737 and 10 mg/kg CsA; and days 4–10: 25–50 mg/kg ABT-737 (mostly 35 mg/kg) and 10 mg/ml CsA. Thereafter, mice continued to receive 10 mg/kg CsA until death. In the initial phase after irradiation and T cell transfer, some of the already weakened animals did not well tolerate ABT-737 treatment and about 25% of animals died during the first few days after transplantation. Accordingly, ABT-737 dose was adjusted to the condition of individual animals. Tregs were depleted on day 14/15 by DT administration. Mice were monitored daily and severity of GVHD was assessed non-blinded by a score consisting of (a) posture/activity, (b) fur texture, (c) skin integrity, and (d) body weight loss, as previously described in Ref. (29).

### *In Vivo* Mixed Lymphocyte Reaction

For the “*in vivo* MLR” experiment, F1 (B6xCBAF1) recipient mice received anti-CD154 antibodies (2 mg) 6 h before tail vein injection of 30 × 10^6^ B6.FoxP3-GFP splenocytes (in the medium as described above for DST). The following 3 days mice received one daily injection of ABT-737/vehicle and CsA. Spleens were harvested and analyzed on day 4.

### Hemisplenectomy

For hemisplenectomy, mice were anesthetized by isoflurane inhalation, pain treated with buprenorphine, and treated with cefazolin (antibiotic). The abdomen was opened by midline incision, and two ligations were placed around the inferior vascular pedicle and the middle of the spleen, before half of the spleen was removed inferior to the intrasplenic ligation. The abdominal wall was closed with continuous suture.

### Statistics

Statistical analysis was performed with GraphPad Prism software. Unpaired two-tailed Student’s *t*-test was used to assess statistical differences between groups. IC_50_ values were calculated using a log(inhibitor) vs. (normalized) response model (variable slope) for *in vitro* ABT-737 sensitivity analysis. Survival in Kaplan–Meier survival curves was compared using log-rank test. In animal experiments, each data point represents results from one single mouse, whereas in *in vitro* experiments, one data point represents the mean of technical replicates (usually triplicates). *P* < 0.05 was considered significant, *P < 0.05; **P < 0.01; ***P < 0.001, and n.s., not significant.

## Results

### Bcl-2/Bcl-xL Inhibition Results in Relative Treg Enrichment

**Figure 1:**
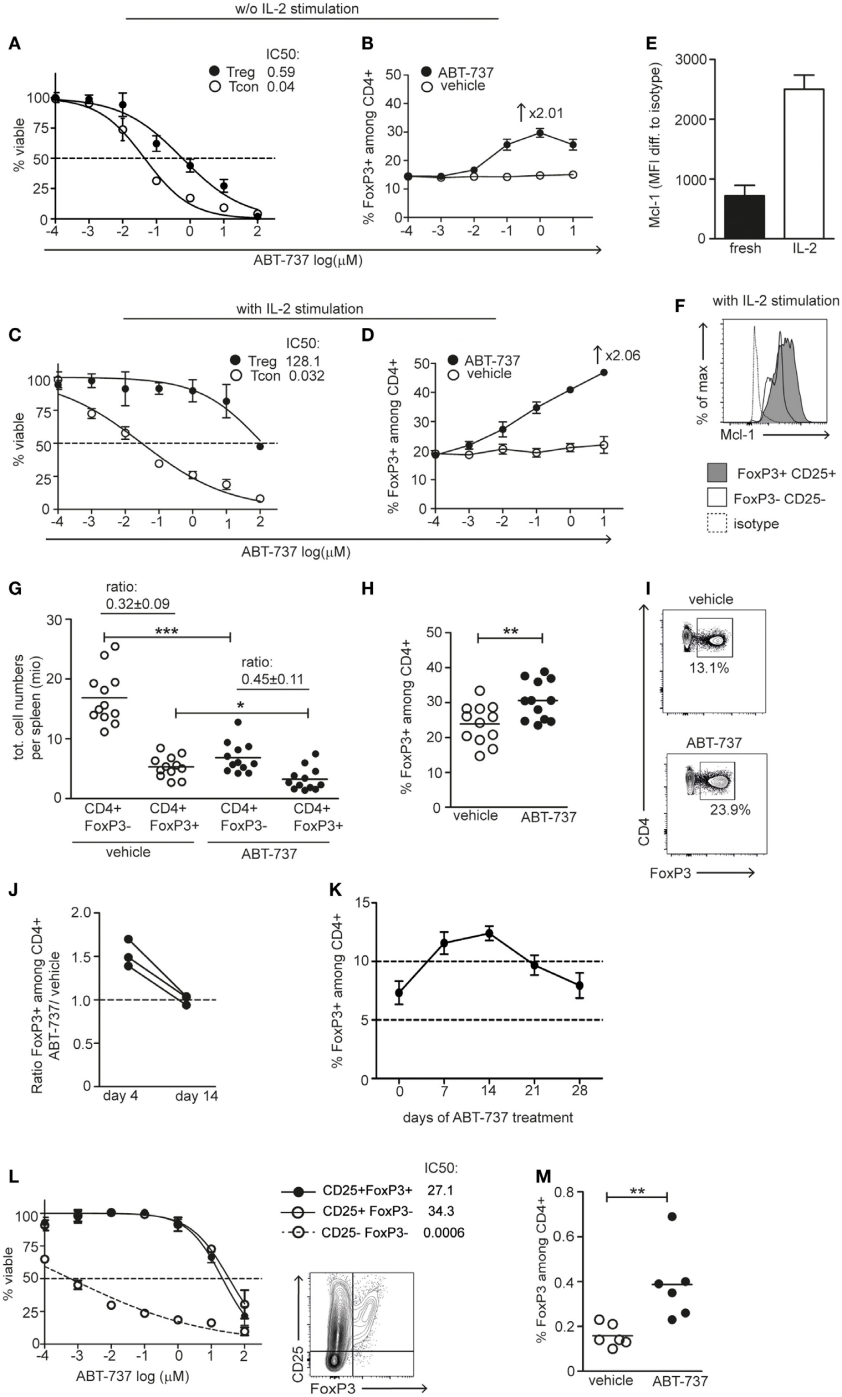
**Naïve and activated Tregs are resistant to Bcl-2/Bcl-xL blockade**. **(A,B)** Freshly isolated FoxP3-GFP splenocytes or **(C,D)** after 3 days of IL-2 stimulation were used for *in vitro* experiments. **(A,C)** Cells were incubated for 12 h with the indicated concentrations of ABT-737. The number of viable CD4^+^FoxP3^+^ (black) and CD4^+^FoxP3^−^ (white) cells per well was assessed by FACS and normalized to corresponding vehicle controls. Curve represents a log(inhibitor) vs. normalized response curve. **(B,D)** The percentages of FoxP3^+^ cells among CD4^+^ cells were assessed after 12h ABT-737/vehicle incubation. *In vitro* titration experiments were performed two to four times, representative experiments are shown. **(E)** Quantification of median expression of Mcl-1 in fresh and IL-2 stimulated (3 days) CD4^+^CD25^+^FoxP3^+^ splenocytes. **(F)** Representative FACS plot showing Mcl-1 expression in Tregs (gray) and Tcons (white) upon IL-2 stimulation in same experiment. **(G–I)** FoxP3-GFP mice were treated with vehicle or ABT-737 i.p. for 3 days prior to harvesting. **(G)** Total counts of CD4^+^FoxP3^−^ and CD4^+^FoxP3^+^ cells per spleen are shown. The ratio corresponds to CD4^+^ FoxP3^+^/FoxP3^−^ of total cell counts in vehicle and ABT-737 treated animals. **(H)** The percentage of FoxP3^+^ cells among CD4^+^ cells. Pooled data of three independent experiments, *n* = 12/group. **(I)** Representative FACS plots of viable CD4^+^ splenocytes in vehicle and ABT-737 treated animals. **(J)** Mice were treated for 3 days with ABT-737 or vehicle and the next day hemisplenectomized for analysis of half spleen, 10 days later the other half spleen was analyzed. The percentage of FoxP3^+^ cells among CD4^+^ population upon ABT-737 treatment was normalized to vehicle-treated mice, *n* = 3/group. **(K)** Percentage of FoxP3^+^ cells among peripheral blood CD4^+^ cells during 4-week ABT-737 treatment, *n* = 4. **(L)** Tregs were activated in a mixed lymphocyte reaction with CD25-depleted B6.FoxP3-GFP responders, CD3-depleted CBA stimulators, and anti-CD154 antibodies for 24 h, and additional IL-2/TGF-β for 3 days. After 12 h incubation with ABT-737 or vehicle, viable cell counts were assessed by FACS and normalized to corresponding vehicle controls. Curve represents a log(inhibitor) vs. response curve. Representative FACS plot of cells after stimulation. One of two independent experiments is shown. **(M)** B6.FoxP3-GFP splenocytes were transferred to F1 (B6xCBA) recipients under anti-CD154 costimulation blockade and treated with ABT-737 or vehicle and CsA for 3 days. Spleens were analyzed on day 4 by FACS. The percentage of transferred FoxP3^+^ cells among all CD4^+^ cells is shown. Pooled data of two independent experiments, *n* = 6/group. All data represent mean ± SEM, *P < 0.05, **P < 0.01, ***P < 0.001, *t*-test was used to assess differences between treatment groups.

To investigate the proapoptotic effect of ABT-737 on different lymphocyte subpopulations, we assessed cell viability after overnight incubation of freshly isolated splenocytes with ABT-737 *in vitro*. Tregs displayed a consistent survival advantage over Tcons, as reflected in a 13.2× higher IC_50_ value (Figure 1A). As a result, ABT-737 treatment led to a relative enrichment of Tregs *in vitro* (from 14.5 to 29.7% Tregs among CD4^+^ cells with increasing ABT-737 concentrations, Figure 1B). This resistance is consistent with the fact that the ABT-737 resistance factor Mcl-1 is of predominant importance for Treg survival (18). Comparison of the protein expression levels of the various antiapoptotic Bcl-2 family members and proapoptotic Bim showed very similar expression of all factors among FoxP3^+^CD25^+^ Treg and FoxP3^−^CD25^−^ Tcon populations, with the only exception of Bcl-xL (Figure S1 in Supplementary Material). After stimulation with IL-2, the resistance of Tregs increased further (approx. 3300× higher IC_50_ value than Tcon), leading to a Treg enrichment up to 47% (Figures 1C,D). This increased resistance to ABT-737 correlated to a strong upregulation of Mcl-1 under the effect of IL-2 (Figures 1E,F).

The relative resistance of Tregs to the proapoptotic effect of ABT-737 was confirmed *in vivo*. Naïve mice were treated for 3 days with a total of five injections of ABT-737. This resulted in moderate lymphopenia as reflected in a reduced number of CD4^+^FoxP3^−^ Tcons in the spleen. Absolute Treg numbers were also reduced upon ABT-737 treatment, but to a lesser extent than Tcon (Figure 1G) (22, 23). Thus, Bcl-2/Bcl-xL inhibition led to a significant higher percentage of Tregs among CD4^+^ cells in comparison to vehicle controls (Figures 1H,I). To follow the enrichment of FoxP3^+^ cells among CD4 T cells in the spleen of the same mouse, hemisplenectomy was performed after 3 days of ABT-737 treatment. A 1.53-fold enrichment of Tregs compared to vehicle-treated animals was observed. FACS analysis of the remaining half of the spleen 10 days later showed equal percentage of FoxP3^+^ cells in the two groups; thus, Treg enrichment was rapidly reversed after therapy discontinuation (Figure 1J). Furthermore, we observed that this enrichment was transient also under continuous treatment with ABT-737, with a peak at day 14 and with a return to baseline levels after 28 days (Figure 1K). Thus, Bcl-2/Bcl-xL inhibition resulted in a relative, temporary enrichment of Tregs *in vitro* and *in vivo*.

### Antigen-Stimulated Tregs Are Resistant to Bcl-2/Bcl-xL Inhibition

Blockade of the CD40-CD154 costimulatory pathway is a widely used approach in experimental tolerance protocols, and these protocols often promote Treg expansion and depend on their suppressive activity (30–32). Thus, we wished to investigate the impact of selective Bcl-2/Bcl-xL inhibition on survival of Tregs under such conditions of concomitant antigenic stimulation and anti-CD154 blockade. We stimulated Tregs *in vitro* from CD25-depleted naïve splenocytes with allogeneic cells in the presence of CD154 blocking antibody (MR1) and TGF-β/IL-2 cytokines. ABT-737 incubation revealed high resistance of alloantigen-activated Tregs, similar to the previously observed resistance of activated CD25^+^ Tcons (IC_50_ value 36,000× higher than in CD25^−^ Tcons) (Figure 1L) (21).

To investigate the properties of activated Tregs *in vivo*, we took advantage of an “*in vivo* MLR model.” B6.FoxP3-GFP splenocytes were transferred into F1 (B6xCBA) recipients under costimulation blockade with anti-CD154 antibodies, followed by ABT-737 or vehicle treatment for 3 days. Low-dose cyclosporine A (CsA) was combined with ABT-737 to prevent TCR–calcineurin–NFAT1-dependent upregulation of Bcl-2A1 (A1) in Tcons and thus preventing resistance of Tcons upon antigenic stimulation (21). ABT-737 treatment resulted in moderate lymphopenia and in a 2.4-fold enrichment of donor-derived Tregs (Figure 1M). Taken together, antigen-stimulated Tregs were resistant to Bcl-2/Bcl-xL inhibition and exposure to ABT-737 resulted in a shift of the balance between Tregs and Tcons in favor of the Treg compartment.

### Treg-Dependent Alleviation of GVHD upon ABT-737 Treatment

**Figure 2:**
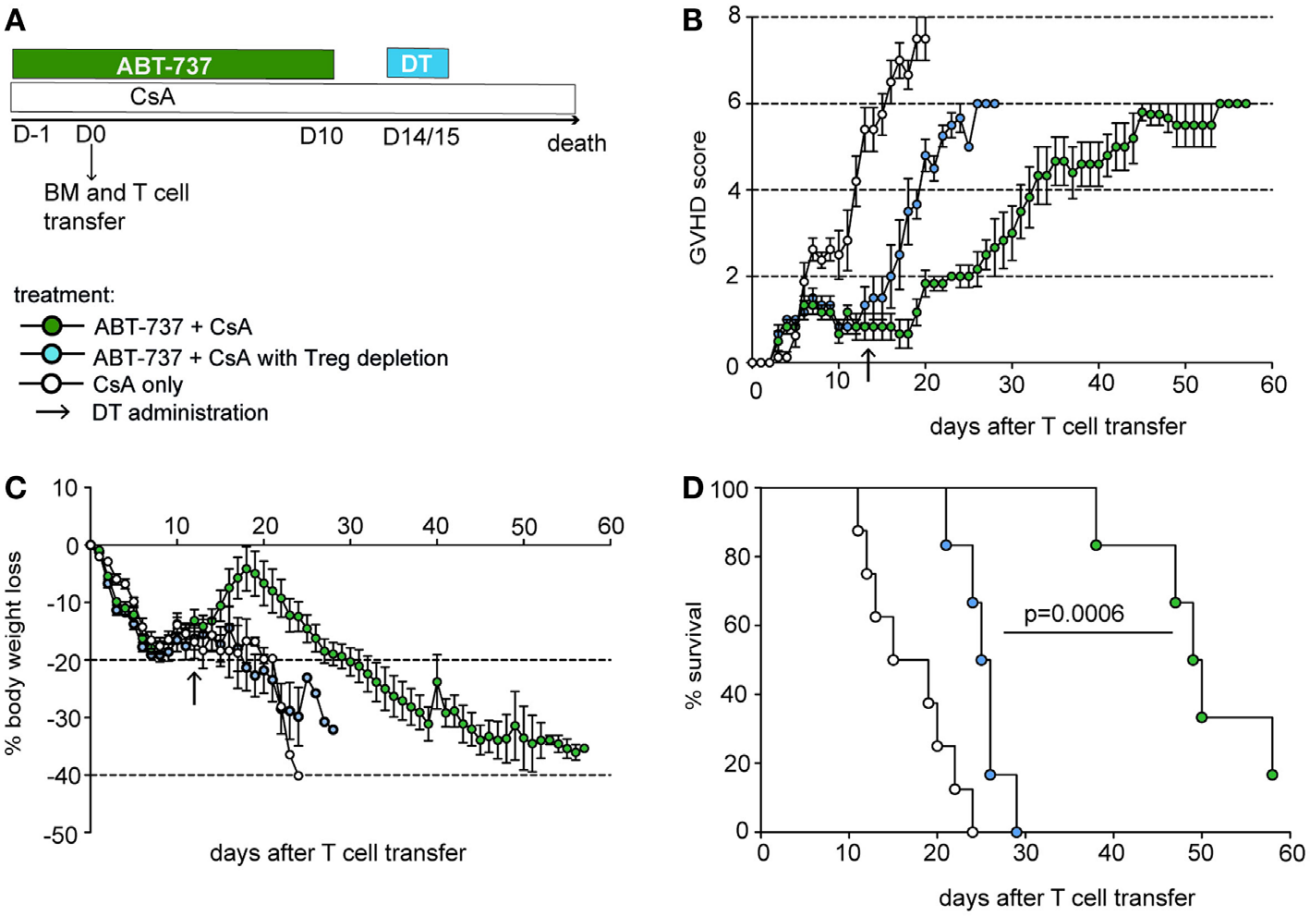
**ABT-737 treatment alleviates GVHD in a Treg-dependent manner**. **(A)** Experimental setup for GVHD experiment: F1 (B6xCBA) mice received DEREG bone marrow cells and T cells after lethal irradiation on day 0 and treatment from day −1 until day 10 with ABT-737 and CsA (blue and green) or CsA only (white) until death. Tregs were depleted by DT administration on day 14/15 (blue). **(B)** GVHD score, **(C)** body weight loss, and **(D)** survival, *n* = 6–8/group. All data represent mean ± SEM, difference between treatment groups was assessed with a log-rank test. Data from one out of two independent experiments are shown.

Regulatory T cells are recognized for alleviating the course of GVHD, and Treg therapy has been successfully tested in this condition (5). Therefore, for the first functional application of our concept, we aimed to investigate whether ABT-737 mediated Treg enrichment has a favorable impact on GVHD. Recipient F1 (B6xCBA) mice were lethally irradiated and reconstituted with allogenic B6.DEREG bone marrow and T cells. DEREG mice express a FoxP3-driven primate DT receptor, which allows selective Treg depletion by DT administration (25). Body weight and a GVHD score consisting of activity/posture, fur texture, skin integrity, and weight loss was assessed daily. Mice were either treated with combined ABT-737 and low-dose CsA or CsA alone, starting 1 day before cell transfer and continued for 10 days (Figure 2A). GVHD onset was about 10 days after transplantation in control mice treated only with CsA, and the disease progressed to lethality within 3 weeks (Figures 2B–D). ABT-737 treated animals presented a markedly attenuated course of GVHD. Due to the severity of the disease, mice were not bled in order to directly show relative enrichment of Tegs upon ABT-737 treatment in this setting. However, Tregs were integral in mediating the positive effects of treatment, as Treg depletion at day 14 after transplantation induced a rapid onset of GVHD [median survival time (MST) 49.5 vs. 25.5 days in Treg depleted group, *P* < 0.001]. None of the ABT-737 treated animals developed skin lesions, and thus, they did not reach the maximal GVHD score. In conclusion, ABT-737 substantially attenuated GVHD across full MHC barriers and this effect was mediated by Tregs.

### ABT-737 Treatment Leads to Treg-Mediated Donor-Specific Hyporesponsiveness

**Figure 3:**
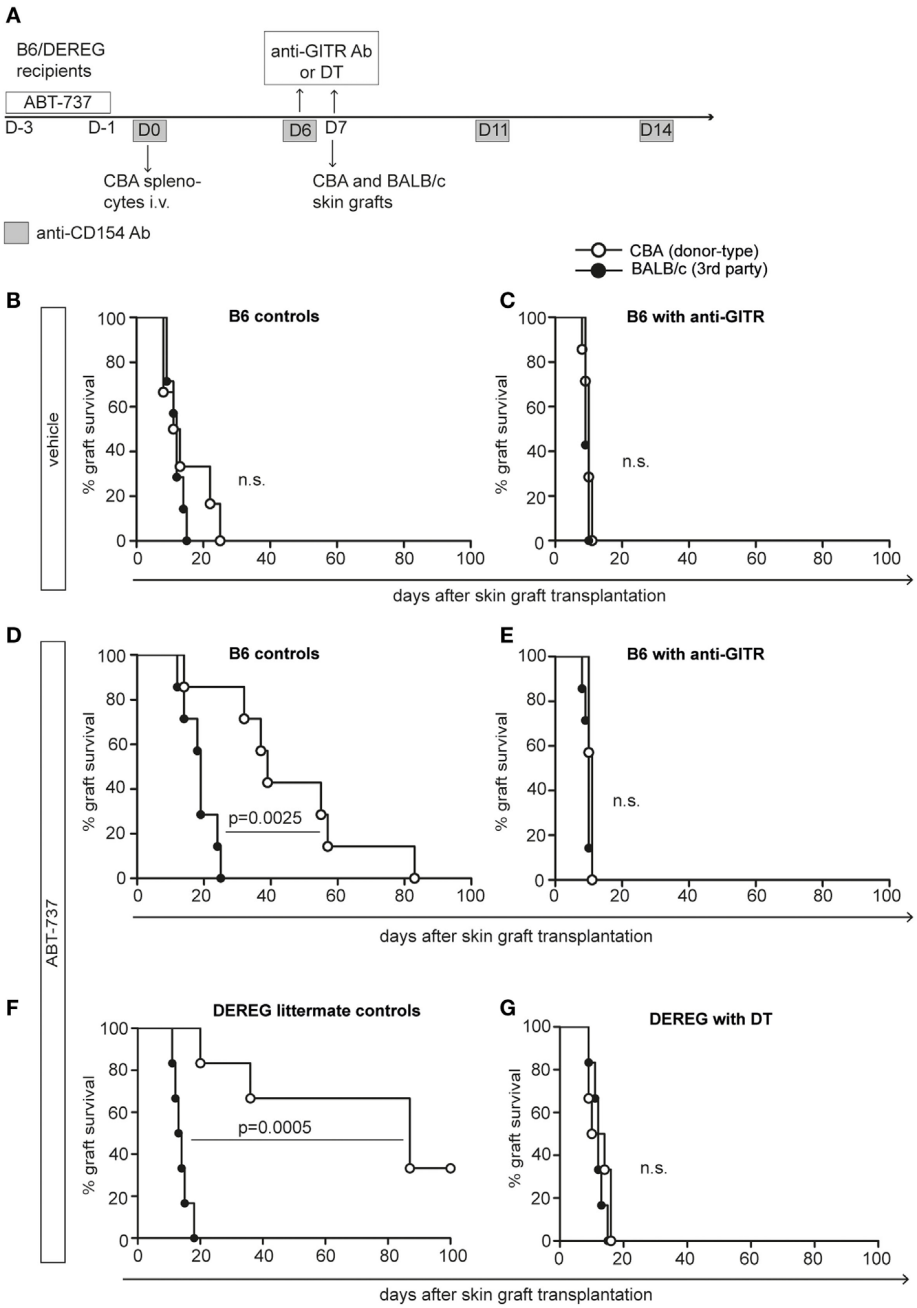
**ABT-737 induces Treg-mediated donor-specific hyporesponsiveness**. **(A)** Experimental setup for DST experiment: B6 or DEREG mice were treated with ABT-737 or vehicle for 3 days prior transfer of CBA splenocytes on day 0 and anti-CD154 antibody treatment on days 0, 6, 11, and 14. On day 7, mice received CBA (donor-type) and BALB/c (third party) skin grafts, and graft survival was monitored until rejection. **(B)** Survival of CBA (white) and BALB/c (black) grafts after vehicle treatment or **(D,F)** with ABT-737 treatment. **(C,E)** Tregs were blocked with anti-GITR antibodies the day prior skin grafting (day 6) in B6 recipients or **(G)** by DT injection (day 6/7) in DEREG recipients. *n* = 7/group, differences between treatment groups were assessed with log-rank test. **(B–G)** derived from independent experiments (data from two out of three independent experiments are shown).

To investigate whether this immunomodulatory effect was antigen-specific, we took advantage of an established model of full MHC-mismatched skin transplantation after DST under costimulation blockade (33). B6 or B6.DEREG-recipient mice received donor-type splenocytes (CBA) under the effect of anti-CD154 antibody (Figure 3A). This induction therapy leads to deletion/anergy of donor-specific Tcons, favors the generation of Tregs, and results in transient donor-specific hyporesponsiveness (34). We combined this protocol with a 3-day ABT-737 treatment prior to i.v. splenocyte injection. Seven days after DST, recipient mice received donor-type (CBA) and third party control (BALB/c) skin grafts. In this stringent strain combination, the original DST protocol (vehicle group) did not significantly prolong skin graft survival (Figure 3B). However, the short additional treatment with ABT-737 was sufficient to markedly prolong donor-type skin graft survival up to more than 100 days in some recipients (MST 39/87 days for ABT-737 and 12/13 days for vehicle, Figures 3D,F). Notably, this effect was donor-specific and not due to generalized immunosuppression, as demonstrated by the only slightly delayed rejection of third party grafts. To assess the importance of Tregs in ABT-737-induced donor-specific hyporesponsiveness in this model, we depleted Tregs by DT injection in DEREG recipients or inhibited their function by GITR (glucocorticoid-induced TNFR family-related gene) ligation the day prior to skin transplantation (35). In absence of a functional Treg compartment, all skin grafts were rapidly rejected, and no difference between donor-type and third party grafts was observed (Figures 3C–G). Thus, although ABT-737 might have additional Treg-independent effects influencing graft survival, we show here that the ABT-737-induced hyporesponsiveness was critically dependent on Tregs, and the transient alteration of the Treg/Tcon balance after ABT-737 treatment was sufficient to create a strong tolerogenic milieu.

### Induction of Transplantation Tolerance by ABT-737 Depends on Tregs

**Figure 4:**
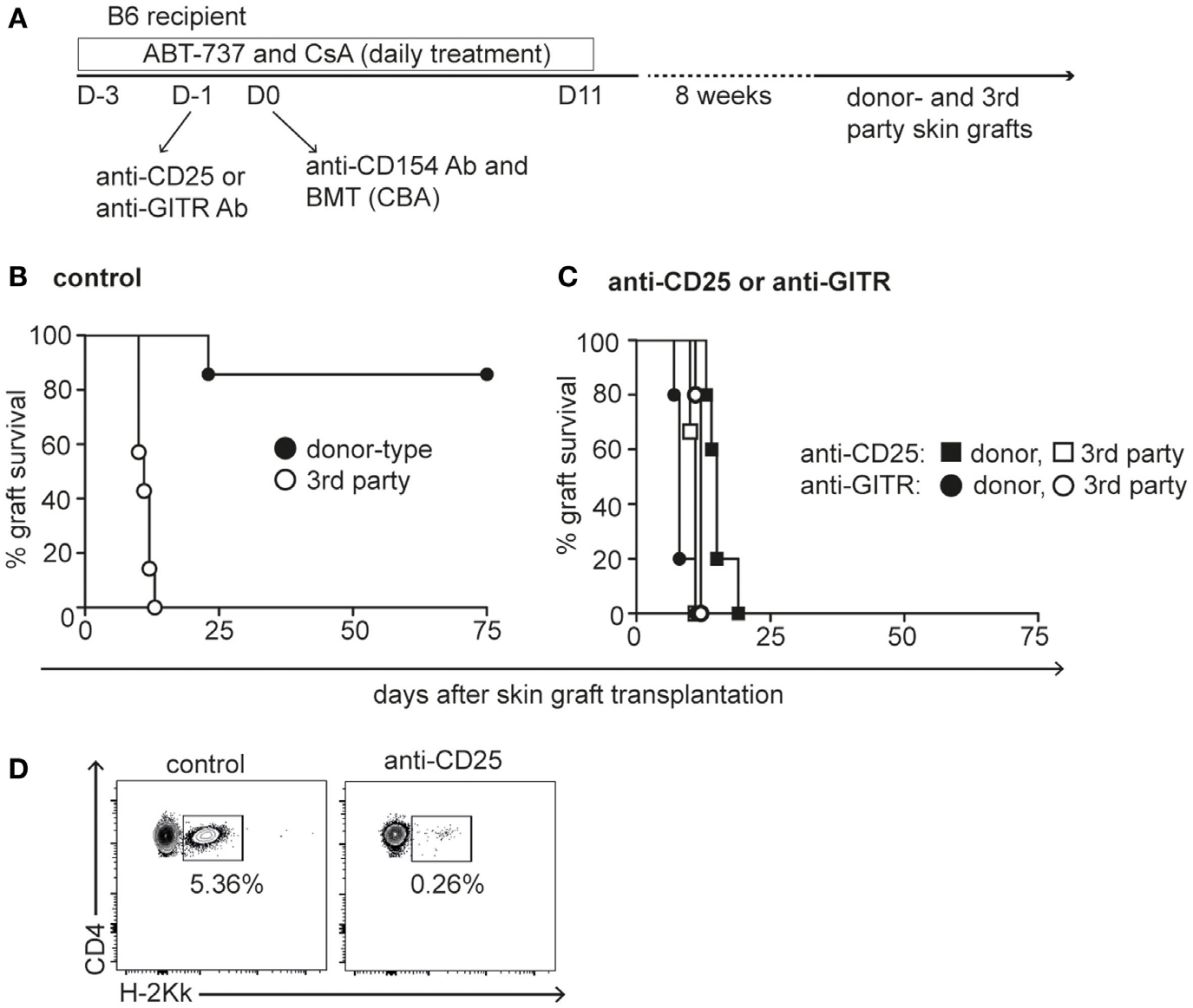
**Tolerance induction by ABT-737 requires Tregs**. **(A)** Experimental setup of mixed chimerism tolerance protocol: B6 recipients were treated with five injections of ABT-737 and CsA during the 3 days prior transplantation of CBA bone marrow cells on day 0. Anti-CD154 antibodies were administered 6 h before bone marrow transplantation, and ABT-737/CsA treatment was continued until day 11 after transplantation. Tregs were blocked by anti-GITR antibodies (circles) or depleted by anti-CD25 antibody administration (squares) at day −1. Eight weeks after transplantation, mice received CBA donor-type (black) and BALB/c third party (white) skin grafts and survival of grafts was monitored until rejection. **(B)** Graft survival of control group and **(C)** of Treg blocked/depleted groups, *n* = 7/group. **(D)** Representative FACS plots of peripheral CD4^+^ cell chimerism in control mouse and Treg depleted mouse 2 months after bone marrow transplantation. Tolerance protocol shown in **(B)** was repeated several times and Treg blockade/depletion by anti-GITR and anti-CD25 once each.

A transient state of Treg-mediated donor-specific hyporesponsiveness might represent an ideal environment to promote engraftment of allogeneic bone marrow cells. We previously showed that a 2-week conditioning treatment with ABT-737, low-dose CsA, costimulation blockade (anti-CD154), and transplantation of 25 × 10^6^ bone marrow cells resulted in stable mixed lymphohematopoietic chimerism and complete deletion of donor-reactive CD8^+^ cells in a fully MHC-mismatched strain combination (Figure 4A) (28). Grafting of donor-type (CBA) and third party control (BALB/c) skin grafts to B6 recipients 8 weeks later confirmed stable donor-specific immunological tolerance in most animals (Figure 4B). We hypothesized that ABT-737 mediated Treg enrichment is a critical factor for tolerance induction in this myelosuppression-free protocol. To test this hypothesis, Tregs were either depleted with anti-CD25 antibody or functionally inhibited by GITR ligation the day prior to BMT. This antibody treatment indeed precluded the establishment of tolerance to skin grafts in all animals, because mixed chimerism induction was hampered in the absence of a functional Treg compartment (Figures 4C,D). Thus, the transient tolerogenic milieu caused by Treg enrichment through Bcl-2/Bcl-xL inhibition was required to induce mixed chimerism without myelosuppressive conditioning.

## Discussion

A dysregulated balance between regulatory and effector cell populations is often involved in overshooting immune activation, such as in transplant rejection, autoimmunity, or allergies. Thus, the achievement of functional dominance of Tregs over Tcons is a major aim in a wide range of clinical conditions. Direct *in vivo* manipulation of the Treg/Tcon balance represents an alternative to Treg therapy in order to achieve Treg-mediated tolerance, and this can be achieved by the selective reduction or functional inhibition of Tcons, while preserving the Treg population. To reach this goal, pathways that are differentially regulated among these two cell populations need to be identified and pharmacologically manipulated. For example, cellular metabolism reflected in differential mTOR activity has been proposed to be such a pathway (36), and low-dose IL-2 administration selectively favored expansion of Tregs (37).

In the present study, we propose the intrinsic apoptosis pathway as promising target to exploit the immunomodulatory potency of Tregs. On a functional level, antiapoptotic Mcl-1 is the predominant survival factor in Tregs, and loss of Mcl-1 results in drastic reduction of Treg numbers. In contrast, loss of Bcl-2 or Bcl-xL does neither result in a relative reduction of Treg numbers nor loss of function (18). Thus, the relative importance of Mcl-1 and Bcl-2/Bcl-xL varies among Tregs and Tcons. According to a previous study, Tregs display a slightly differential expression pattern in apoptosis-related genes than Tcons, which might account for differences in sensitivity to cytotoxic stress (38). There, the biggest difference was found in Bcl-xL expression, a finding that we confirmed on protein level. Despite the facts that Bcl-xL is bound with high affinity by ABT-737 and at the same time seems to be redundant for Treg survival, it may confer resistance toward ABT-737-mediated apoptosis. In fact, it has been shown that increased Bcl-xL expression in lymphocytes confers resistance to ABT-737-mediated killing (39). In the present study, we pharmacologically exploited these differences in the regulation of the intrinsic apoptosis pathway among Tregs and Tcons. Combined inhibition of antiapoptotic Bcl-2 and Bcl-xL induced a functionally important Treg enrichment *in vivo*. This concept was tested in very stringent immunological models: short-term Bcl-2/Bcl-xL inhibition was sufficient to substantially alleviate GVHD, to inhibit allogeneic immune responses against fully MHC-mismatched skin grafts and to allow bone marrow engraftment without myelosuppression in a mixed chimersm model. The tolerogenic effect of ABT-737 was only transient, but in combination with BMT, this approach allowed the induction of stable tolerance across full MHC barriers. Importantly, activated Tregs were even more resistant to ABT-737 than naïve Tregs, and these activated Tregs are presumably responsible for the donor-specificity observed in skin graft experiments. This fact offers the opportunity to generate an antigen-specific tolerogenic environment with broad clinical implication for immunomodulatory therapies. In transplantation medicine, donor-specific immunosuppression would represent an important step toward the prevention of rejection without the risks for infections and cancers.

Evasion of apoptosis is one of the hallmarks of cancer, as Bcl-2 factors are often overexpressed in malignant cells. Thus, large efforts are taken for developing highly selective Bcl-2 family inhibitors. To date, little is known about apoptosis regulation in human T cell subsets. For clinical translation of the proposed immunomodulatory concept, closer research on human cells will be needed, but tailored Bcl-2 inhibition is likely to translate into clinical therapies. Several Bcl-2 inhibitors have already entered clinical trials for various cancers and recently, a phase I study with ABT-199 in systemic lupus erythematosus patients has been completed (40–42). Bcl-2 inhibitors showed a favorable toxicity profile so far. The present study might stimulate further research on the effect of these inhibitors on T cells subsets directly in patients and importantly, it might draw attention to potentially unwanted side-effects in cancer patients with regard to a tumor immunology point-of-view by changing the Treg/Tcon balance on the short-term.

## Author Contributions

SG, TF, and PC designed experiments and wrote the paper. SG performed all experiments, analyzed the data, and prepared the figures. NB helped with *in vitro* experiments and JC with skin grafts and hemisplenectomy. TW and AB provided scientific input. TF supervised the project.

## Conflict of Interest Statement

The authors declare that the research was conducted in the absence of any commercial or financial relationships that could be construed as a potential conflict of interest.
