# Supplementary material for: Distinctive Expression of Bcl-2 Factors in Regulatory T Cells Determines a Pharmacological Target to Induce Immunological Tolerance
Source: Front Immunol. 2016 Mar 1;7:73. doi: 10.3389/fimmu.2016.00073 (PMC4771729; doi:10.3389/fimmu.2016.00073)
Supplement: Supplementary file 1 [file Image_1.PDF]

Supplementary Material

Distinctive expression of Bcl-2 factors in regulatory T cells determines a pharmacological target to induce immunological tolerance

Sarah Sharon Gabriel, Nina Bon, Jin Chen, Thomas Wekerle, Andrew Bushell, Thomas Fehr, Pietro Ernesto Cippà*

*** Correspondence:** Pietro Ernesto Cippà: pietro.cippa@usz.ch

Supplementary Figure 1

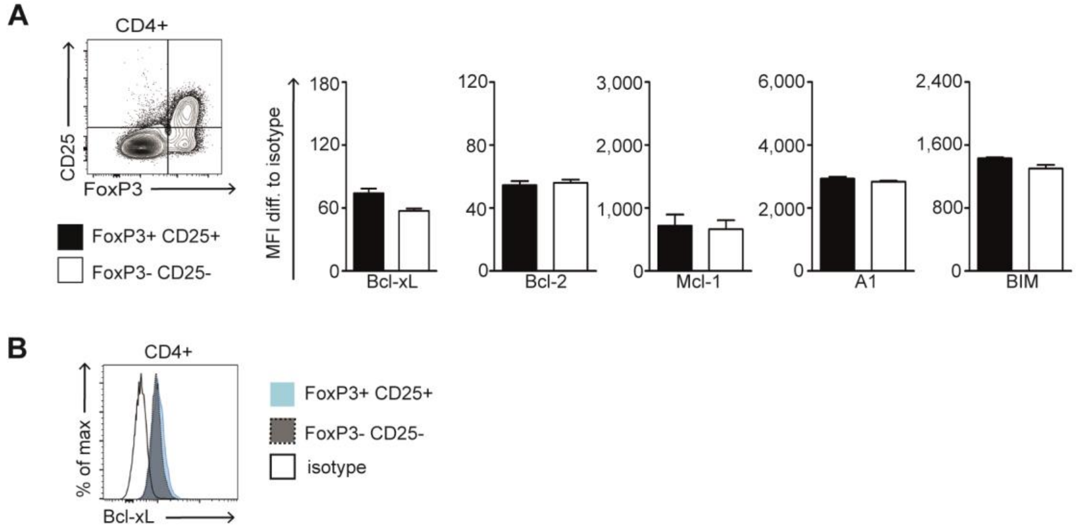

Supplementary Figure 1.

(A) Representative FACS plots showing CD25 and FoxP3 expression in viable CD4+ cells. Quantification of median expression of Bcl-xL, Bcl-2, Mcl-1, A1 and Bim in $CD4^+CD25^+FoxP3^+$ cells (black) and $CD4^+CD25^-FoxP3^-$ cells (white). **(B)** Representative FACS plot of Bcl-xL expression in Tregs (blue) and Tcon (grey). The stainings were performed in triplicates and repeated three times, one representative experiment is shown. Data represent mean ± SEM of technical replicates.
